# Emerging functional connectivity differences in newborn infants vulnerable to autism spectrum disorders

**DOI:** 10.1038/s41398-020-0805-y

**Published:** 2020-05-06

**Authors:** Judit Ciarrusta, Ralica Dimitrova, Dafnis Batalle, Jonathan O’Muircheartaigh, Lucilio Cordero-Grande, Anthony Price, Emer Hughes, Johanna Kangas, Emily Perry, Ayesha Javed, Jill Demilew, Joseph Hajnal, Anthony David Edwards, Declan Murphy, Tomoki Arichi, Grainne McAlonan

**Affiliations:** 1 Centre for the Developing Brain, School Biomedical Engineering and Imaging Sciences, King’s College London, St Thomas’ Hospital, London, SE1 7EH United Kingdom; 2 Dept. of Forensic and Neurodevelopmental Sciences, Institute of Psychiatry, Psychology and Neuroscience, King’s College London, Denmark Hill, London, SE5 8AB United Kingdom; 3 MRC Centre for Neurodevelopmental Disorders, King’s College London, London, SE1 1UL United Kingdom; 4 South London and Maudsley NHS Foundation Trust, London, United Kingdom; 5 Department of Bioengineering, Imperial College London, London, SW7 2AZ United Kingdom

**Keywords:** Neuroscience, Autism spectrum disorders

## Abstract

Studies in animal models of autism spectrum disorders (ASD) suggest atypical early neural activity is a core vulnerability mechanism which alters functional connectivity and predisposes to dysmaturation of neural circuits. However, underlying biological changes associated to ASD in humans remain unclear. Results from functional connectivity studies of individuals diagnosed with ASD are highly heterogeneous, in part because of complex life-long secondary and/or compensatory events. To minimize these confounds and examine primary vulnerability mechanisms, we need to investigate very early brain development. Here, we tested the hypothesis that brain functional connectivity is altered in neonates who are vulnerable to this condition due to a family history of ASD. We acquired high temporal resolution multiband resting state functional magnetic resonance imaging (fMRI) in newborn infants with and without a first-degree relative with ASD. Differences in local functional connectivity were quantified using regional homogeneity (ReHo) analysis and long-range connectivity was assessed using distance correlation analysis. Neonates who have a first-degree relative with ASD had significantly higher ReHo within multiple resting state networks in comparison to age matched controls; there were no differences in long range connectivity. Atypical local functional activity may constitute a biomarker of vulnerability, that might precede disruptions in long range connectivity reported in older individuals diagnosed with ASD.

## Introduction

Autism spectrum disorders (ASD) are increasingly common, with current prevalence estimated to be 1 in 59 children^1^. Infants with a family history of ASD are known to have greater vulnerability to developing ASD compared to infants without family history. The higher likelihood is estimated to be 2-fold for cousins, 4.8-fold for half siblings and between 9.3- and 17-fold for full siblings^2,3^. This increased but variable likelihood may be explained because the underlying etiology comprises a highly heterogeneous interaction of genes and the environment, within every individual^4–6^. In addition, ongoing genetic and environmental mechanisms cause secondary and/or compensatory changes in the brain across the lifespan. Such complexity may explain why the results of studies exploring pathophysiology in individuals with a diagnosis of ASD are often contradictory or inconclusive. It suggests that what is inherited or acquired in ASD is not fixed, but may better be conceptualized as a “vulnerability” to brain dysmaturation^7^. In line with this, we now know that, even as early as toddlerhood, parental intervention can alter the developmental trajectory of infants with family history of ASD^8,9^. Therefore, to truly understand the biology of ASD, we must start by examining how family history might make the brain vulnerable to dysmaturation, before postnatal events cloud the interpretation of results.

Evidence from small rodent models suggests that the pathophysiological consequences of genetic (and/or environmental) risks for ASD are present from very early in development and include atypical synaptic mechanisms and altered neural activity^10–14^. These preclinical studies consistently report an increase in spontaneous synchronous activity in the brain of rodents carrying risk genes for ASD around what would be the equivalent time of birth in humans^15^. This aberrant activity is first seen in the primary sensory cortices^12^ and then later involves higher order regions such as the prefrontal lobe^13^. As early synchronous activity in local circuits is thought to be an essential step in the establishment of functional connectivity which helps to shape the topography of brain networks^16,17^, its disruption could potentially have far-reaching consequences for brain development. However, no-one has tested whether vulnerability to ASD due to family history is associated with aberrant activity patterns in the perinatal time window as suggested by results from studies of early brain development in rodents carrying risk genes for ASD.

Dynamic changes in the blood oxygen level dependent (BOLD) signal during functional magnetic resonance imaging (fMRI) permits assessment of neural activity across the whole brain at both a local and systems-wide level in humans. Using these methods, individuals with a diagnosis of ASD have been reported to have both under and over connectivity in diverse local networks, as well as in long-range between network connections [see ref. ^18^ for a review]. Although these studies have been important in revealing a central role for functional dysconnectivity in ASD, untangling primary mechanisms from secondary consequences of living with ASD, has not been possible. This is of particular relevance as in contrast to the aforementioned studies in individuals with an established diagnosis of ASD, the use of functional connectivity to understand typical and atypical early brain development is a much newer endeavor^19^. Recent studies of functional connectivity in typically developing infants show that connectivity between voxels increases in the somatosensory cortex from 30 to 40 weeks PMA^20^ and long range connectivity between distal regions of the brain increases later, from 4 to 9 months postnatally^21^. However, it is unknown how local and long-range connectivity matures in the perinatal window in neonates with a family history of ASD, and therefore at higher likelihood of atypical development compared to peers without a family history.

Here, we used fMRI to explore the pathophysiological signatures of early life vulnerability to ASD. Newborn infants were categorized into two groups, with family history of ASD (FAM+) and without family history of ASD (FAM−). Participants in the FAM+ group had a first-degree relative with ASD, and therefore were at a higher likelihood of atypical development, while neonates in the FAM− group had no first-degree relatives with ASD. We studied the synchronicity/local functional connectivity of emerging brain networks using ‘Regional Homogeneity’ (ReHo) analysis^22^; as this approach has been shown to be sensitive to differences in short-range functional activity in older children and adults with ASD^23–25^. We then calculated long-range functional connectivity between 92 distinct brain regions using distance correlations^26^. Based on the preclinical literature and current knowledge about typical functional connectivity development^23,24^, we hypothesized that synchronous activity/local connectivity would be higher in the FAM+ than FAM− group. We also hypothesized that because long-range connectivity has yet to begin to mature at this age^24^, there would be no group differences on this measure.

## Material and methods

A total of 33 newborn babies with a first degree relative with ASD were recruited as part of the EU-AIMS Brain Imaging in Babies (BiBs) study approved by the South London, National Research Ethics Committee. A detailed medical and psychiatric history was taken from the mother of each potential participant, as well as a comprehensive screen to exclude major maternal medical conditions during pregnancy or delivery complications affecting infant health. After applying strict criteria to exclude fsMRI data corrupted by head motion during acquisition (further details in *Data processing* section) and cases with incidental intracerebral findings, the final study group consisted of 20 FAM+. 20 age matched controls (FAM−) without any family history for neurodevselopmental disorders or any other incidental findings were selected from the entirely independent developing human connectome project (dHCP) cohort. The characteristics of the sample are described in detail in Table 1. Further demographic details about maternal depression scores, maternal age, maternal years of education, maternal body mass index and baby’s birth weight are provided in Supplementary Figure 1. Shapiro–Wilk test confirmed all demographic variables were normally distributed in both groups, however we report median and range because of the small sample size. Within the final dataset for analysis, of the FAM+ group, 16 had a first degree relative with ASD and 4 had a first degree relative with both ASD and ADHD. All infants in the final sample had a normal appearance on structural MR images (as reported by a Neonatal Neuroradiologist) and did not have a history of birth asphyxia or significant clinical difficulties in the neonatal period.

**Table 1:** FAM+ and FAM− group characteristics.

	FAM+ (*n* = 20)		FAM− (*n* = 20)	
	median	range	median	range
Birth GA (weeks)	39.64	[34.43–41.14]	40.00	[34.14–42.00]
Birth weight (kg)	3.42	[2.80–4.20]	3.32	[1.57–4.25]
Birth head circumference (cm)	33.50	[32.00–37.00]	34.50	[30.00–36.00]
Scan PMA (weeks)	42.36	[40.00–44.86]	42.36	[39.57–44.71]
Scan weight (kg)	4.20	[2.90–5.20]	3.55	[2.90–4.50]
Scan head circumference (cm)	36.60	[34.00–39.00]	35.85	[34.00–37.60]
Male/Female	14/6	—	13/7	—

Gestational age (GA) at birth, weight in kilograms (kg) at birth, head circumference in centimetres (cm) at birth, post menstrual age (PMA) at the day of scan, weight in kilograms (kg) at the day of scan, head circumference (ci.) in centimetres (cm) at the day of scan, and total of male and female subjects are described for each group.

### Data acquisition

Written informed consent was taken from the parents of all participants prior to data acquisition on scan date. All scans were acquired with the same protocol on the 3T MRI scanner in the clinical research facility located at the Neonatal Intensive Care Unit at St Thomas’ Hospital, London. High temporal resolution resting state fMRI data was acquired in a 3T Philips Achieva scanner (Best, NL) with a custom made 32 channel neonatal head coil and imaging system (Rapid GmbH, Riedstadt DE)^27^, using a multi-slice echo planar imaging (EPI) sequence with multiband (MB) excitation (MB factor 9) and parameters; TR: 392 msec, TE: 38 msec, spatial resolution: 2.15 mm isotropic, total time: 15 min 1.6 s (2300 volumes)^28^. High-resolution T1 and T2 weighted images were acquired for registration and clinical reporting purposes. All data was acquired during natural sleep following feeding. Infants were wrapped still and securely using a vacuum-evacuated mattress (Med-Vac, CFI Medical Solutions, Fenton, MI, USA) and hearing protection was applied (molded dental putty (President Putty, Coltene Whaledent, Mahwah, NJ, USA) in addition to adhesive earmuffs (MiniMuffs, Natus Medical, Inc., San Carlos, CA, USA)).

### Functional data pre-processing

Data pre-processing was carried out using a pipeline specifically developed and optimized for sources of artifacts inherent to neonatal fMRI data, including uncontrolled head motion and developmental differences in brain configuration and tissue contrast. Images were processed using tools implemented in the FMRIB Sofware Library (FSL)^29^. The framewise displacement with root-mean square matrix calculation (FDRMS) of each volume (from the sum in all directions of the rigid body motion between consecutive volumes) was calculated for all 2300 acquired volumes. Acquired data was cropped to 1600 continuous volumes with the least amount of motion for further analysis. Subjects with high framewise displacement (FDRMS > 0.5 mm) in more than 80 volumes out of 1600 total volumes (5% of cropped dataset) were excluded from analysis. The final dataset comprised 20 FAM+ infants and 20 FAM− infants. Independent component analysis (ICA) as implemented in MELODIC (Model-free FMRI analysis using Probabilistic Independent Component Analysis [PICA, v3.0]) was used to identify signal artifacts (motion, MB and cardiac / respiratory) and to train a classification algorithm for automatic identification of these sources of noise, which were then regressed out from the data using the FSL FIX tool^30,31^. Next, we run topup to estimate the warp embedding field distortions caused by susceptibility artifacts^32^. We used this warp, combined with each subject’s anatomical T2, to non-linearly transform each subject’s functional data to a 41-week-old neonatal template^33^ using FSL registration tools.

### Functional data analysis

Pre-processed functional data in standard space was used to run temporal concatenated group ICA with a fixed dimensionality of 25 components^34^.

Voxel-wise regional homogeneity was assessed in native space by calculating the Kendall coefficient of concordance between the BOLD contrast time-series of a given voxel with its 26 adjacent neighbors using AFNI 3dReHo tool^35^. The calculated Kendall’s Tau (τ) was then converted to Pearson’s *r* (*r* = sin(*πτ*/2)) and transformed into Fisher’s z-scores for standardizing the values across the sample for statistical comparison^36^. ReHo data were then warped into standard space using the same method and warp that was used to register the functional data to the 41-week-old neonatal template^33^.

Anatomical parcellation of each subject was based in the neonatal version of AAL parcellation^37,38^ adapted to the high-resolution dHCP neonatal template space^39^. The parcellation was then adapted to the specific 41 week old neonatal template used in this study^33^. Long-range functional connectivity between the resulting 92 regions (90 cortical and subcortical regions together with right and left cerebellum) was characterized using multivariate distance correlation^26^.

### Statistical analysis

General linear model and dual regression methods implemented in FSL were used to identify group means and test differences for each network identified with group ICA.

Selected ICA networks were binarized (threshold *p* < 0.01) and used as masks to extract ReHo values of each network. Cohen’s d was used to compare ReHo values between networks. Permutation testing as implemented in FSL’s Randomize (v2.1)^40^ with false discovery rate (FDR) correction for multiple comparisons was used to compare regional homogeneity z-score maps and distance correlation matrices between groups controlling for gestational age at birth, PMA at scan, sex and motion outliers.

## Results

### Resting state networks

**Fig. 1:**
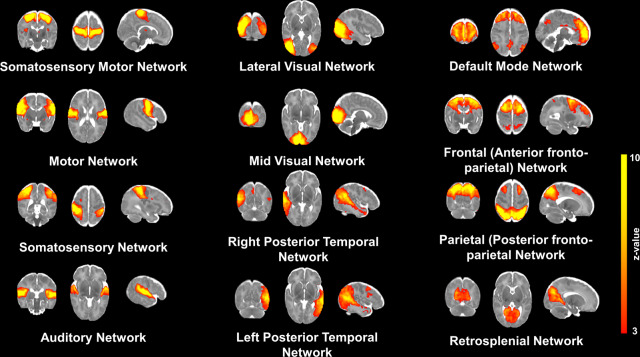
Resting State Networks in the Neonatal Brain. Coronal, axial and sagittal examples of 12 independent components extracted with probabilistic ICA are overlaid on a neonatal term-equivalent template. The spatial representation of the RSN was threshold to a z-statistic between 3 and 10. The 12 ICA networks depicted correspond to somatosensory/motor (paracentral), motor, somatosensory, auditory, lateral visual, mid visual, left and right posterior temporal (temporo-parietal), default mode network (DMN), Frontal (anterior fronto-parietal), Parietal (posterior fronto-parietal) and retrosplenial RSNs.

The topography of resting state networks (RSN) was characterized using probabilistic independent component analysis (ICA)^34^. Group independent component analysis revealed twelve distinct networks which corresponded with previously identified RSNs^41,42^. The somatosensory/ motor (paracentral), motor, somatosensory, auditory, lateral visual, mid visual, left and right posterior temporal (temporo-parietal), default mode network (DMN), Frontal (anterior fronto-parietal), parietal (posterior fronto-parietal) and retrosplenial networks that were observed for the entire group are represented in Fig. 1. Dual regression analysis revealed no differences between groups in the spatial distribution of the RSNs.

### Network regional homogeneity

**Fig. 2:**
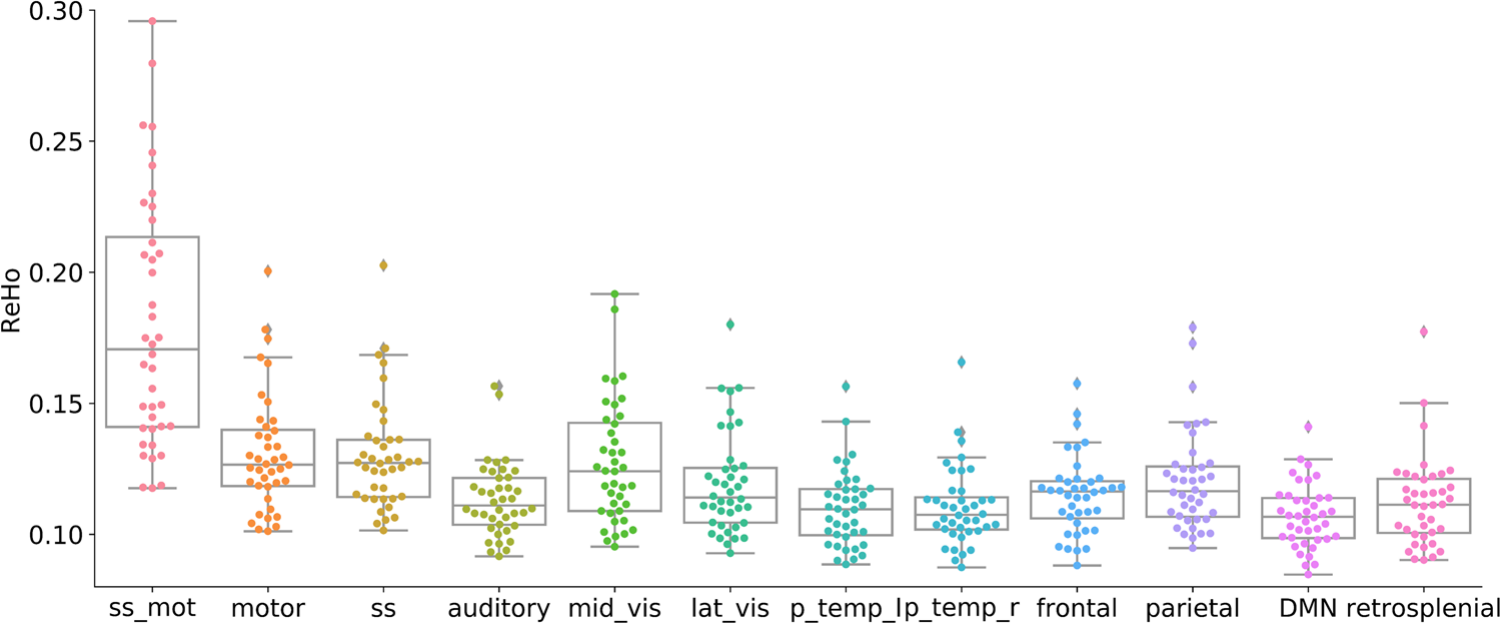
Within RSN local functional connectivity. The median of each subject for the somatosensory/motor (ss_mot), motor, somatosensory (ss), auditory, mid visual (mid_vis), lateral visual (lat_vis), left (p_temp_l) and right posterior temporal (p_temp_r), the default mode network (DMN), Frontal (anterior fronto-parietal), Parietal (posterior fronto-parietal) and retrosplenial RSNs is represented in a data point within a boxplot for each RSN.

The median ReHo value for each RSN was calculated for each subject. We observed no significant differences between groups for the median ReHo values. Across all subjects, ReHo median values in the somatosensory/motor (paracentral) network were significantly higher than any other network, with the greatest difference observed in comparison to the DMN (*d* = 2.1, corrected *p* < 0.01). Somatosensory and Motor networks also showed significantly higher ReHo in comparison to the Auditory, Posterior Temporal right and left, Frontal, DMN and retrosplenial networks, with greatest differences observed relative to the DMN (*d* = 1.34, corrected *p* < 0.01). Mid Visual network showed significantly higher ReHo relative to the posterior temporal network left and right (*d* = 0.9, corrected *p* < 0.01) and the Frontal network showed higher ReHo compared to the DMN (*d* = 0.8, corrected *p* < 0.01) (see Fig. 2).

### Within network short-range functional connectivity

**Fig. 3:**
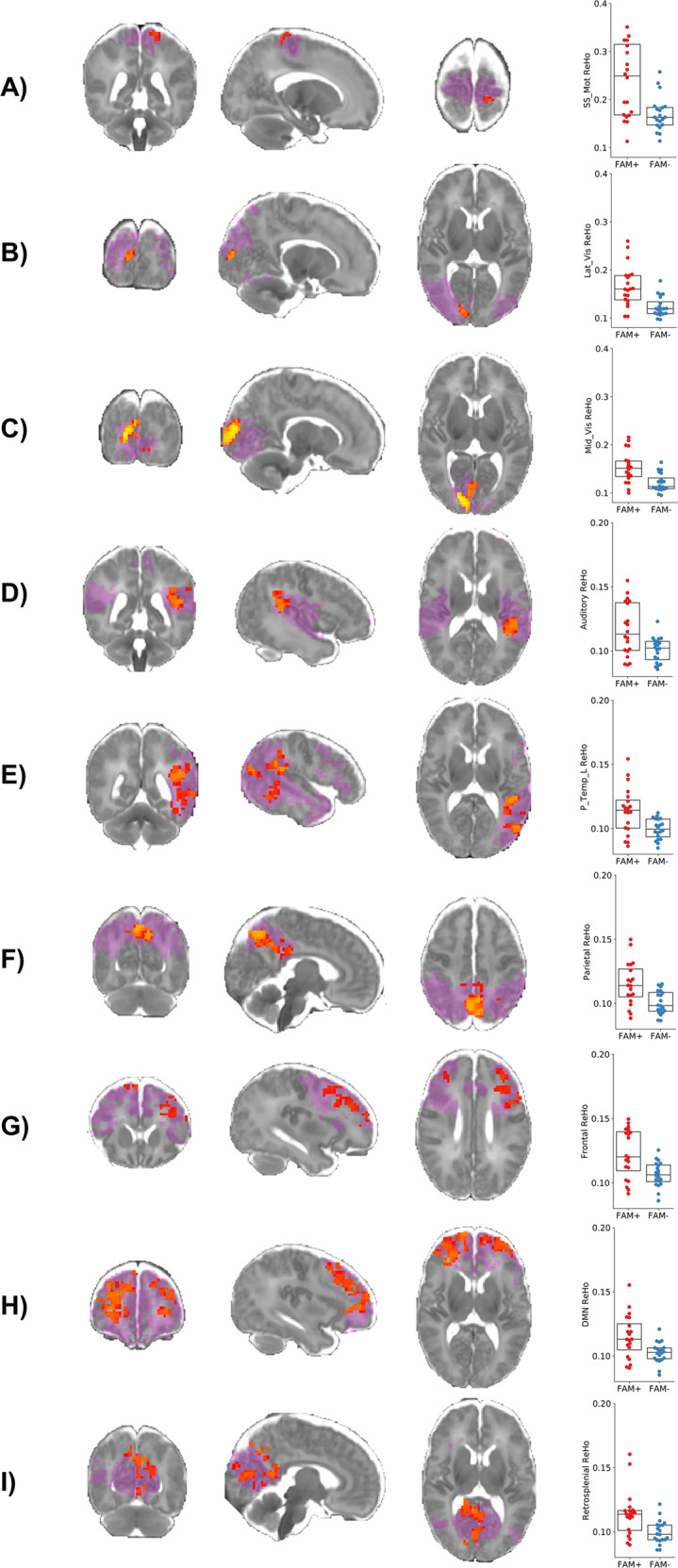
Local functional connectivity differences between FAM+ and FAM− sample. The binarized mask of each resting state network (lilac) is overlaid on a T2 weighted 42 PMA week template. The results of a non-parametric t-test comparing ReHo Fisher z-scores between groups are depicted in red-yellow. The somatosensory/motor, lateral visual, mid visual, auditory, left posterior temporal, parietal, frontal, DMN and retrosplenial RSNs showed extensive clusters of significantly higher ReHo in the FAM+ sample after false discovery rate (FDR) correction.

A voxel-wise non-parametric two sample unpaired T- test of the Fisher’s z-scores (with age at birth, age at scan and gender as covariates and with false discovery rate correction, *p* < FAM−0.001) demonstrated ReHo values were significantly higher in the FAM+ group compared to the FAM−. These differences were located within the somatosensory/motor (paracentral), lateral visual, mid visual, auditory, posterior temporal left, frontal, parietal, DMN and retrosplenial networks. There were no networks where the FAM+ group had significantly lower ReHo values than the FAM− group (see Fig. 3). The T-statistic maps threshold between *t* = 2.6 and *t* = 5 for each of the nine networks with significantly different ReHo values are provided in Supplementary Figure 2.

Additional analysis retaining only the infants that were born at term equivalent age and excluding the infants that were born at preterm equivalent age showed the same significant differences between the FAM+ and FAM− subjects. There was no material difference in findings aside from the observation that the spatial extent of significant clusters was qualitatively larger; all results were in the same regions.

### Long-range functional connectivity

**Fig. 4:**
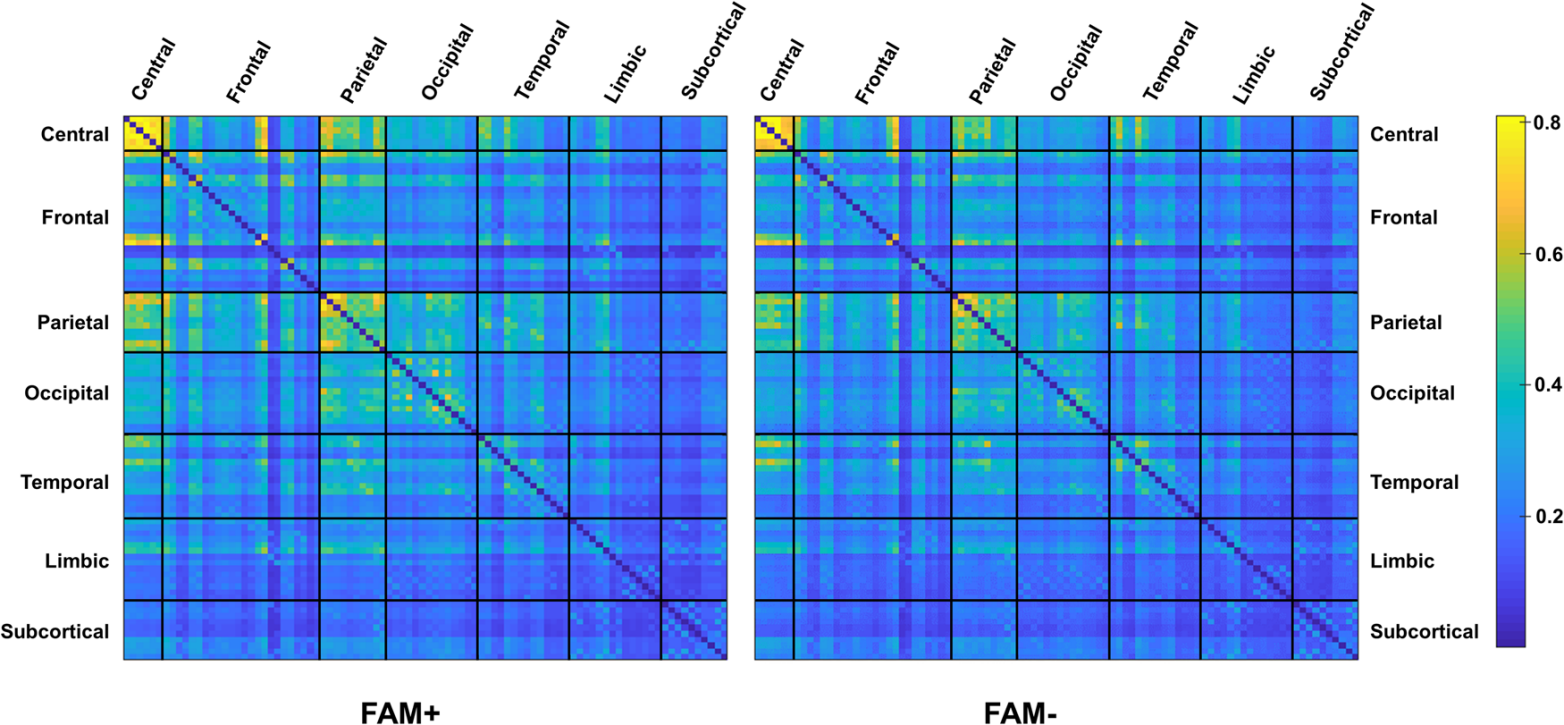
Long-range connectivity in the FAM+ and FAM− sample. The distance correlation between 92 anatomical regions of interest is depicted for the FAM+ in the right and the FAM− in the left. Strongest correlations can be observed in yellow within the central and parietal regions.

For both groups, we observe a tendency towards higher DC values between central regions parieto-parietal, parietal and frontal regions, particularly the prefrontal and orbitofrontal areas, as well as between parietal and supplementary motor cortex. There were no significant differences in DC values for any of the ROI pairs between the 2 groups (see Fig. 4).

## Discussion

A peak in synchronous activity in brain circuits maturing in the perinatal period is a fundamental feature of neural circuit development^17,43^. Here, we used a combination of high temporal resolution fMRI, an optimized age-specific image processing pipeline, ReHo analysis and ICA, as well as distance correlations analysis, to characterize short- and long-range functional connectivity in resting state networks in human infants shortly after birth. We show that newborn infants with family history of ASD, have higher local connectivity levels than control infants, but no difference in long range connectivity. The highest values of ReHo were found in the sensory networks of both groups, which are known to mature during this perinatal period.

### Mechanisms underpinning atypical local functional activity

In the mammalian brain, synchronous activity commences around the equivalent period to mid-gestation in humans. Mouse studies have found that spontaneous activity is largely modulated by synaptic GABAergic^44,45^ and glutamatergic signaling^46,47^. In mice, highly synchronous oscillations slowly disappear as inhibitory properties mature^48^. The shift of GABA from excitatory to inhibitory is essential for experience dependent mechanisms to commence and foster further maturation of neural networks^49^. This process occurs in the mouse somatosensory and visual cortex within the first two weeks of postnatal life (equivalent to the last seven weeks of human fetal development)^50,51^; but the transition occurs later in higher order regions such as the frontal lobe^52^. Although the timing of these events in the human brain is uncertain, recent post mortem studies in humans suggest a similar profile as GABAergic interneurons do not migrate to the frontal lobe until the first 5 to 7 months after birth^53^. Given the critical role for many ASD genes in the regulation of synaptic mechanisms^54,55^, we suggest that genetic risks for ASD in the FAM+ group contribute to anomalies in local activity/functional connectivity patterns observed in this cohort. Evidence suggests genetic regulatory processes of diverse origin appear disrupted in ASD consistently affecting neuronal activity^56,57^.

### Implications for brain development in childhood

#### Structure and Function

The abnormalities observed in our FAM+ group at birth are in line with atypical connectivity reported in older children and adults with a diagnosis of ASD. ReHo differences in ASD are highly heterogeneous across different developmental stages, showing over-connectivity earlier on followed by under-connectivity^24,25,58^. The widely varied maturational trajectories in individuals with ASD^23,59,60^ may reflect heterogeneity that is established in early development, which is then influenced by on-going environmental exposures and/or compensatory mechanisms across the life-span.

#### Cognition and Behavior

Early anatomical and functional alterations in lower-order brain networks also fit with atypicalities in visual, auditory and tactile information processing in older individuals with ASD^61^; and now recognized in DSM-5^62^. These, unusual sensory features are now thought to appear in advance of ‘higher-order’ clinical abnormalities. Infants with family history of ASD at 3 months have been reported to have a higher prevalence of unusual sensory behaviors^63^ and at 9 months they have enhanced visual search which predicts autistic symptom severity at 24 months^64^. Similarly, toddlers with ASD who are classified as ‘sensory reactive’, have less mature language abilities^65^. It is therefore plausible that the atypical activity patterns observed in ‘lower-order’ networks within our FAM+ sample might represent and ‘imbalance’ in the sequence and/or trajectory of lower and higher-order skill development.

However, although differences in sensory regions were prominent, differences did involve other networks. Thus, it is possible that this activity profile could be a generalized characteristic which acts across the whole-brain of neonates with a family history of neurodevelopmental disorders but is most evident in sensory behaviors because these are developing at the time of examination.

### Building blocks of long-range functional activity

The strong alterations in local connectivity in the FAM+ group contrast with the absence of any significant differences in long-range connectivity between the FAM+ and FAM− groups. Functional integration between distant regions is known to mature during the first postnatal year in typically developing infants^21^. Thus, disruptions to local activity whether experience dependent in sensory regions or spontaneously driven in higher order regions might precede atypical interaction with other brain regions later in development^66^, causing what has been termed a “developmental disconnection”^67^. Consistent with this, pioneering studies of young infants with family history of ASD have used functional data to explore network dynamics and have shown that reduced network efficiency at 6 months is associated with ASD like traits at 24 months^68^. A similar study in older infants found that higher functional connectivity between distal regions was linked with repetitive behavior at 24 months^69^. Taken together, our results indicate atypical local connectivity may precede the long-range connectivity anomalies associated with ASD that appear later in life and suggests a potential vulnerability mechanism which predisposes to the patterns observed in older age groups.

### Idiosyncratic activity patterns and outcome

Although we plan to follow the development of our participants into early childhood, we emphasize that this study was *not* designed to provide a predictive test of a binary diagnostic outcome. Rather, the goal of this study was to test a biological hypothesis regarding vulnerability mechanisms for ASD. Although our findings arise from groups defined by their differences in vulnerability, in this cross-sectional study, we cannot exclude the possibility that the activity differences observed within the FAM+ group may reflect resilience rather than risk, as not all the group will have developmental difficulties. To properly address this issue, we require longitudinal studies and going forward we plan to follow-up these infants to formally assess if they later develop a neurodevelopmental disorder. Future work will determine how connectivity patterns change with age and we will take a dimensional approach that captures future neurodevelopmental traits (in addition to binary diagnostic outcomes). We suggest that the results observed here describe an atypical mechanism that features high local functional connectivity in the perinatal period. That is, the neonatal activity ‘finger-print’ across functional networks of an individual with family history of ASD may eventually map to an individual’s phenotype. This is important, as we know from animal studies that altering the environment/experience can shape the development of these networks. We also know that altering the early human environment, for example through parent intervention, can improve outcomes in ASD^8,9^. Therefore, an individual’s functional connectivity pattern may someday help to provide a personalized set of targets to optimize outcomes in infants vulnerable of developing neurodevelopmental disorders.

### Limitations

Data were acquired in this study using a dedicated neonatal MR imaging system^27^ and an optimized high temporal resolution fMRI sequence and data analysis pipeline. This allowed us to address specific challenges inherent to neonatal fMRI studies and avoid sources of potential bias in the analysis. Whilst it is known that ReHo can be confounded by partial volume errors leading to the inclusion of non-gray matter tissue^25^, we found there were no significant differences in gray matter volume between groups; and we were further able to mitigate this effect through the relatively high spatial resolution of our fMRI data (2.15 mm isotropic) and through spatial normalization to an age-specific structural atlas^33^.

As head motion during fMRI data acquisition is known to cause signal artifact and significantly affect estimates of functional connectivity^70^, we applied extremely strict data exclusion criteria for head motion in this study: analyzing only 1600 continuous volumes of the total 2300 acquired in each dataset to ensure that absolute head displacement was less than 0.5 mm for 95% of the data; and discarding datasets entirely from infants where greater than 5% of the acquired volumes were corrupted by FD > 0.5 mm. Although the total duration of the available timeseries for each infant to was therefore reduced to 10 min 27 s, the very high temporal resolution of the data ensured that our ReHo measurements remained robust^71^. This high sampling rate was also important as it allowed improved removal of physiological noise which can be problematic in neonatal fMRI data as infants have naturally higher breathing and cardiac pulsation rates^72^.

As it was noted in the discussion, the lack of outcome data makes it difficult to ascertain the association between the observed findings with behavior later in infancy. Future studies will aid to disentangle whether the reported atypical activity patterns in newborn infants are associated to typical or atypical behavior.

## Conclusions

We demonstrate for the first time that human neonates with a family history of ASD have increased local functional connectivity within RSNs. The observed connectivity may contribute to early atypical behaviors reported in some toddlers with a family history of ASD and may precede differences in functional connectivity seen in older individuals with ASD. Future studies will examine how functional connectivity characteristics relate to later cognitive development, whether elevated local functional activity predicts individual outcomes in later childhood and whether these can be modulated during early development through interventional strategies.

## Supplementary information

Sup_1. Maternal and Baby Demographics

Sup_2. T statistic for ReHo differences
