# Supplementary material for: Emerging functional connectivity differences in newborn infants vulnerable to autism spectrum disorders
Source: Transl Psychiatry. 2020 May 6;10:131. doi: 10.1038/s41398-020-0805-y (PMC7203016; doi:10.1038/s41398-020-0805-y)
Supplement: Supplementary file 1 — Sup_1. Maternal and Baby Demographics [file 41398_2020_805_MOESM1_ESM.pdf]

Supplementary Figure 1

	FAM+			FAM-				
	median	*range*	*mean(sd)*	*median*	*range*	*mean(sd)*	*t*	*p*
**MATERNAL EPDS**	5	[0-12]	5.05 (3.78)	4	[0-11]	4.6 (2.54)	0.44	0.66
**MATERNAL AGE**	35	[28-46]	35.3 (4.39)	34	[20-39]	32.9 (4.63)	1.68	0.1
**MATERNAL YEARS OF EDUCATION**	24	[16-32]	23.2 (4.38)	21.5	[18-28]	21.9 (2.67)	1.22	0.23
**MATERNAL BMI**	26.8	[19.5-35.4]	27.02 (6.28)	23.6	[17.8-29.4]	23.68 (3.33)	1.8	0.08
**BIRTH WEIGHT (KG)**	3.414	[2.8-4.2]	3.4 (0.46)	3.315	[1.6-4.2]	3.28 (0.62)	0.72	0.48

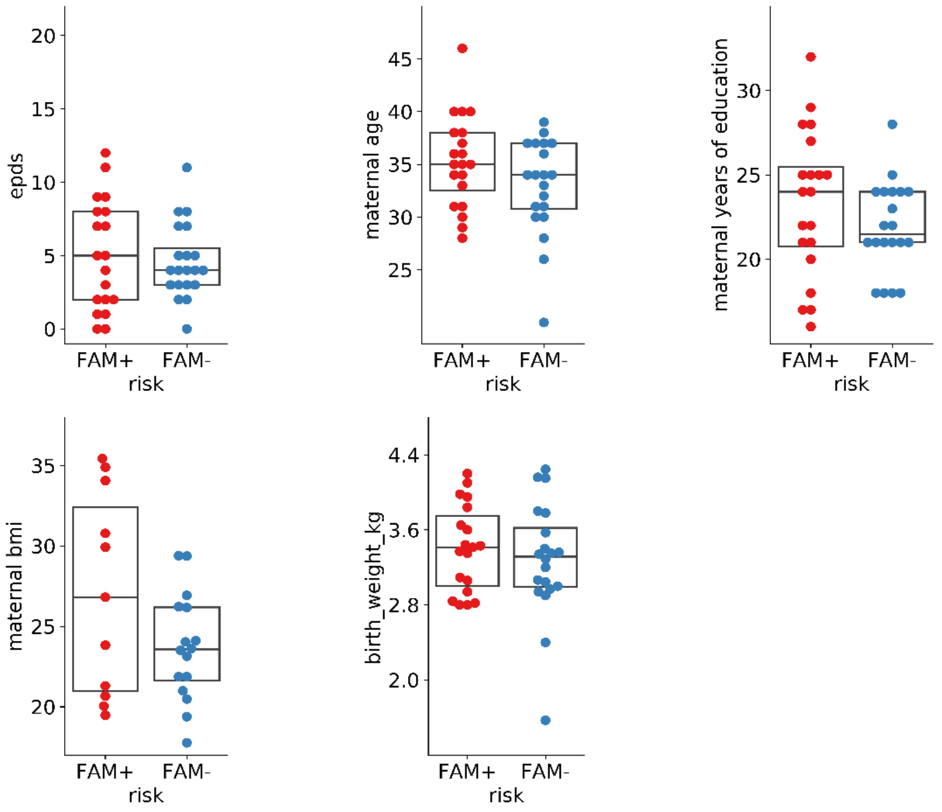

Supplementary Figure 1. Maternal and Baby Demographics. The table shows median, range and mean together with standard deviation for maternal Edinburgh Postnatal Depression Score (EPDS), maternal age, maternal years of education, maternal body mass index (bmi) and baby's birth weight in kilograms (kg) for the group of newborn infants with family history of ASD (FAM+) and without (FAM-). There was no significant difference between groups for any of the variables measured as shown with the t statistic and p-value in the table. The boxplots show the distribution of values for the variables measured in the FAM+ and FAM- newborn infants.
