# Supplementary material for: Emerging functional connectivity differences in newborn infants vulnerable to autism spectrum disorders
Source: Transl Psychiatry. 2020 May 6;10:131. doi: 10.1038/s41398-020-0805-y (PMC7203016; doi:10.1038/s41398-020-0805-y)
Supplement: Supplementary file 2 — Sup_2. T statistic for ReHo differences [file 41398_2020_805_MOESM2_ESM.pdf]

## Supplementary Figure 2

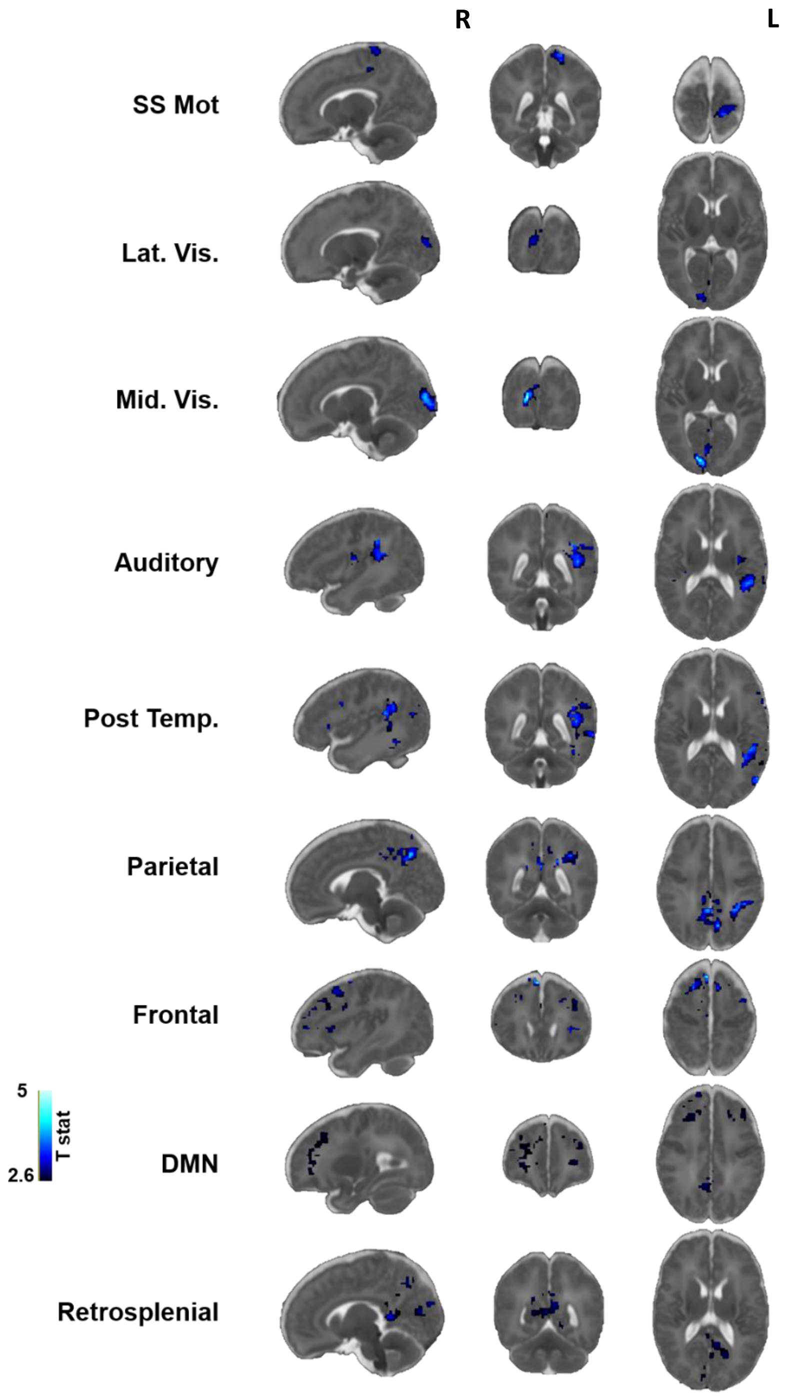


**Supplementary Figure 2. T statistic for Regional Homogeneity differences between groups.** Each row represents a resting state network where significant differences between groups were observed. The T statistic is thresholded to appear only if higher than 2.6 and overlaid on a neonatal T2 template.
